# Using Social Networking Sites for Communicable Disease Control: Innovative Contact Tracing or Breach of Confidentiality?

**DOI:** 10.1093/phe/pht023

**Published:** 2013-08-13

**Authors:** Kate L. Mandeville, Matthew Harris, H. Lucy Thomas, Yimmy Chow, Claude Seng

**Affiliations:** Wellcome Trust Research Fellow, London School of Hygiene and Tropical Medicine; Academic Clinical Lecturer in Public Health, Imperial College London; Consultant in Communicable Disease Control, North West London Health Protection Team, Public Health England; Consultant in Communicable Disease Control, North West London Health Protection Team, Public Health England; Consultant in Communicable Disease Control, North West London Health Protection Team, Public Health England

## Abstract

Social media applications such as Twitter, YouTube and Facebook have attained huge popularity, with more than three billion people and organizations predicted to have a social networking account by 2015. Social media offers a rapid avenue of communication with the public and has potential benefits for communicable disease control and surveillance. However, its application in everyday public health practice raises a number of important issues around confidentiality and autonomy. We report here a case from local level health protection where the friend of an individual with meningococcal septicaemia used a social networking site to notify potential contacts.

## Introduction

Social media refers to ‘activities, practices, and behaviors among communities of people who gather online to share information, knowledge, and opinions using conversational media’. Social media applications include blogs and microblogs such as Twitter, media sharing websites such as YouTube and social networking sites such as Facebook (Safko and Brake, 2009). The latter have attained huge popularity, with more than three billion people and organizations predicted to have a social networking account by 2015 (Thackeray *et al.*, 2012).

There is a growing interest by public health organizations in the use of social media in the dissemination of health information, emergency preparedness and communicable disease control, particularly after the H1N1 influenza pandemic (Jones, 2011; Merchant *et al.*, 2011; Thackeray *et al.*, 2012). For example, the World Health Organisation, Centers for Disease Control and Prevention and Health Protection Agency (HPA) all have Twitter accounts, Facebook pages and videos on YouTube (Jones, 2011). The number of people following the Centers for Disease Control and Prevention’s ‘emergency profile’ on Twitter increased from 65,000 to 1.2 million within a year (Merchant *et al.*, 2011), and the WHO used its Twitter account to issue advice in the wake of the 2011 Japanese earthquake (Jones, 2011). In addition to the dissemination of official information, social media offers a rapid avenue of communication with the public and potential benefits for communicable disease control and surveillance. However, its application in everyday public health practice especially with regard to individual cases raises a number of important issues around confidentiality and autonomy.

We report here a case from a local health protection service, where the friend of an individual with meningococcal septicaemia used a social networking site to inform potential contacts.

## Case Summary

In 2010, a student living in shared accommodation became drowsy with a fever and a widespread rash, and he was admitted to hospital with suspected meningococcal septicaemia. After arrival, the patient’s condition deteriorated, and he was later transferred to intensive care in a coma.

In the UK, there is a statutory requirement for clinicians to notify their local health protection team of a case of probable meningococcal disease. Until 1 April 2013, Health Protection Units (HPU) were the local services of the HPA in England and Wales with responsibility for communicable disease control in their geographical area (these are now part of Public Health England) (Health Protection Agency, 2013). On notification of an index case, local health protection teams co-ordinate activities to reduce secondary spread, communicate public health messages and control outbreaks. For meningococcal disease, these activities include the tracing and risk assessment of close contacts to give antibiotic prophylaxis (Health Protection Agency Meningococcus and Haemophilus Forum, 2012). The contacts at greatest risk of developing disease following a primary case are those living in the same household as the case, and the risk is highest during the first 7 days after a case (Hastings *et al.*, 1997); therefore, earlier prophylaxis is more likely to be effective. Contacts that require chemoprophylaxis should ideally receive this within 24 hours of the diagnosis being made.

As part of the usual response to the notification of a case of probable meningococcal disease, the public health doctor on-call that day identified potential household contacts by phoning a family member and flatmates of the index case. He arranged for chemoprophylaxis to be given to contacts where needed as per national guidance (Health Protection Agency Meningococcus and Haemophilus Forum, 2012). Further health protection actions included contacting the institution where the index case worked to ensure there were no further cases and to provide public health reassurance and advice. The local general practitioners (GP) and Primary Care Trust were also informed for surveillance and communication purposes. The HPA Communication Team was involved at an early stage.

One week after presentation, the HPU was informed by a contact of the index case that the case had other intimate contacts that the family might not have been aware of. With the index case still in a coma, it was not possible to verify this information or obtain contact details. Believing to be taking initiative, and without prior discussion with the HPU, this person had posted a message on the index case’s Facebook ‘wall’ informing three named contacts that the index case had meningitis and telling them to speak to a doctor. Such messages can be read by all ‘friends’ in an individual’s Facebook contact list.

After being notified of the Facebook posting, the HPU team were concerned that such a message did not provide the information to enable close contacts to receive an appropriate risk assessment and could cause unnecessary anxiety amongst those friends and family who were at minimal or no risk. The HPU team requested that the message be modified so that it provided clearer guidance, did not ignite unnecessary concern and became more useful in terms of contact tracing. The modified message, approved by the HPU team, read:

‘On recommendation of the Health Authority, I am contacting people with whom [index case] may have had close recent contact to inform you that XX has been taken ill with possible Meningococcal Disease. You are advised to make contact with the Health Protection Unit on this number xxxx quoting the ID number xxxx’.

One of the named contacts subsequently made contact with the HPU, and a risk assessment of their need for chemoprophylaxis was carried out.

After several days in hospital, the patient made a successful recovery. He was able to inform the HPU that there had been no other close contacts that the HPU had not already identified and risk assessed. Laboratory samples confirmed infection with *Neisseria meningitidis*.

## Case Discussion

This case poses some interesting case management and ethical dilemmas—first, around the use of social media in communicable disease control *per se*, and second, its use in situations where the index case is unable to consent to disclosure of information.

The benefits of social media for communicable disease control are being increasingly recognized. It is already being used for syndromic surveillance, for example in influenza (Ginsberg *et al.*, 2009; Signorini *et al.*, 2011). Local health protection teams traditionally work with organizations such as GP practices, local authorities, schools and universities, to communicate with the public, using methods such as letters or leaflets. Even with technology such as email cascades, these are blunt and not timely instruments for communicable disease control. In certain groups, such as adolescents, students and travellers, there is a need to find ways to communicate risk, identify contacts and implement public health actions that are innovative and better reflect these groups’ lifestyles. By using natural social groupings, social networking sites such as Facebook offer the potential for a highly efficient and effective means of identifying potential contacts and information dissemination for public health purposes. Some social networking sites offer the facility to divide ‘friends’ into different social groups, such as ‘work’, ‘family’ or ‘acquaintances’, thus aiding targeted contact tracing. Moreover, social media offers more stability than traditional communication details such as telephone numbers or addresses. Where the index case is able to work with the public health practitioner, social media offers an alternative means of communication with contacts. This may be particularly useful for diseases where the potential transmission may have been some time ago, for example in tuberculosis or sexually transmitted diseases.

There are, however, a number of concerns surrounding the use of social media. First, there is the potential towards ‘viral’ dissemination of information compared with traditional communication methods such as individual phone calls or letters. Even when carried out carefully, the use of social media has the potential to disseminate information beyond the original target population. If the information is written without public health guidance and is incorrect, this dissemination could have potentially damaging consequences. Second, there is uncertainty over the use of information about the individual for commercial gain by the companies that own the social media websites.

In this situation, the index case was unable to provide informed consent to contact tracing within the relevant period. In such circumstances, there are clear tensions between protecting the confidentiality of the case with the autonomy of contacts potentially at risk of disease. Without any other means to get in touch with relevant contacts, social media may offer a means of communication within the critical window. However, this creates a tension between maximizing the potential contacts reached and not being able to control which contacts are reached. The use of social media is likely to require a more careful assessment of the balance of risks and benefits than the use of traditional media, which may include making greater efforts to obtain consent before posting confidential information.

Although it may appear counter-intuitive, it is unclear whether traditional communication methods confer more protection of an individual’s confidentiality than using social networking sites. Patient identifiable information is routinely excluded from letters to potential contacts from health protection teams as per guidance from the General Medical Council (General Medical Council, 2009); however, contacts may often be able to infer the identity of the index case. From there, it is beyond the control of public health practitioner to contain the spread of rumour and gossip. While the public health practitioner has not directly played a part in that breach of confidentiality, the pursuit of good public health practice will still have led to it. Information dissemination via social networking sites could potentially counter public anxiety more effectively by limiting the initial information transmission to those who know the case. In the case presented here, a pre-existing breach of confidentiality was caused by a lay person, albeit with good intentions, by posting a comment on the Facebook page. However, this raises issues about accountability and responsibility. Is it only public health authorities that can be held accountable to the use and/or misuse of social media for contract tracing, or can a lay person that has caused unnecessary anxiety also be held to account?

Before deciding whether to use social media for communicable disease control, it would seem prudent to weigh the disease characteristics and risk to the public against the social media dimensions and risk to confidentiality. For example, are interventions available to reduce the risk of infection after an exposure and how effective are these? In the absence of an intervention, what is the risk of infection, and the morbidity and mortality from the disease? Will the use of social media necessarily identify the individual case? What is the influence of the social media under consideration? What are the page views/retweeting rates? Who will have access to this information? What are the security settings? If it is decided that the disease and the risk to the population are important enough to warrant the use of social media, some may be more useful than others. For example, Facebook might be more useful to trace household or social contacts, but LinkedIn could be more useful to trace work colleagues.

Finally, if public health practitioners are to use social networking sites as part of their armoury for disease control activities, do we need guidance on how to use them effectively and securely?

## Funding

This work was supported by the Wellcome Trust [grant number 094017 to K.L.M.] and the National Institute for Health Research [to M.H.].

## Conflict of interest

None declared.
